# Deployment Challenges in Electromagnetic Wireless Nanosensor Networks

**DOI:** 10.3390/s25237123

**Published:** 2025-11-21

**Authors:** Amani Bamuqabel, Saoucene Mahfoudh

**Affiliations:** 1 Faculty of Computing and Information Technology, King Abdul Aziz University—KAU, Jeddah 21589, Saudi Arabia; abamuqabel@kau.edu.sa; 2 School of Engineering, Computing, and Design, Dar Al-Hekma University, Jeddah 34801, Saudi Arabia

**Keywords:** wirelessnanosensor network, deployment, energy efficient, topology, coverage, connectivity, terahertz band

## Abstract

Wireless nanosensor networks (WNSNs) are a future technology that will help transform many ideas into reality. Therefore, WNSNs must be properly set up to achieve their intended application goals during the deployment stage. An effective deployment protocol should ensure full coverage of the target area, continuous connectivity between nanosensors, and efficient energy consumption. Since nanosensors are known to have limited energy and computational power, deployment strategies for WNSNs must account for their constrained capabilities and unique properties. In this paper, we review some of existing research on the deployment of WNSNs. Furthermore, we discuss different models that can influence the design of effective deployment strategies for WNSNs.

## 1. Introduction

Wireless nanosensors (NSs) represent a new vision that is expected to change the way many current issues are addressed. NSs possess physical properties and unique behaviors that do not exist in their microscopic-scale counterparts [1,2]. By integrating NSs into wireless nanosensor networks (WNSNs), a new range of innovations and applications is closer to becoming possible [1]. However, for a WNSN to achieve the intended goals of the application it supports, the network must be set up properly and efficiently during the deployment stage. Although deployment is a vital aspect of WNSN research, many challenges remain in designing effective deployment strategies. These challenges stem from the nanoscale nature of NSs. Therefore, directly applying traditional protocols and schemes developed for conventional wireless sensor networks (WSNs) to WNSNs is not a feasible option.

The first major challenge in studying wireless nanosensor networks (WNSNs) is the limited computational capability of nanosensors (NSs). Due to this constraint, NSs can perform only very simple tasks [3,4]. Moreover, they are expected to transmit only small amounts of information encoded in a few bits [5]. Consequently, information exchange within WNSNs requires the use of specialized communication methods. Four approaches have been proposed as suitable for NS communication: molecular, optical, acoustic, and electromagnetic (EM) [6]. While optical and acoustic communications for WNSNs have been discussed in several studies [7,8,9], molecular and EM communications are considered the primary methods suitable for WNSN applications [2].

Molecular communication is defined as exploiting the molecules in the medium to conduct the communication between NSs by encoding the information in the molecules using molecular transceivers [2]. Molecular communication entitles its own channel models, network architecture, and communication protocols, which is not the focus of this paper.

Meanwhile, EM communication describes the processes of transmitting and receiving electromagnetic radiation for the communication between NSs, using novel nano materials [2,10]. In this paper, we focus on WNSNs that employ EM communication as the primary means of information exchange between NSs.

The second challenge that researchers must address is the limited energy of NSs. Since NSs have very limited energy and it is infeasible to replace or recharge their batteries, energy-harvesting systems have been developed to support the perpetual operation of WNSNs. These systems convert various types of ambient energy into electrical energy [11]. However, when NSs rely on such harvesting systems, their available energy fluctuates between surplus and deficit [12], which must be considered when designing solutions for WNSNs [13].

The third challenge is the unusual properties of NSs, which impose several constraints on their capabilities. These constraints can be summarized as follows:

- Graphene antennas: The graphene-based antennas used in NSs are a direct consequence of their extremely small size. Studies have shown that the electromagnetic properties of these antennas, particularly when operating in the Terahertz (THz) band (0.1–10.0 THz), make them the most suitable option for communication between NSs over distances ranging from hundreds to thousands of nanometers [5]. Moreover, the use of the Terahertz band offers several advantages for nanoscale communication:
  1. It enables NSs to transmit and receive information with very large bandwidth, thereby supporting the use of simple communication and medium-sharing mechanisms [4]. Nevertheless, the Terahertz band suffers from very high propagation loss. Combined with the limited energy of NSs, this loss imposes significant limitations on the communication range of NSs [13].
  2. The band offers a high bit rate and short transmission time, which reduce the probability of collisions [14].
- Communication between NSs requires the use of Media Access Control (MAC) protocols to coordinate access to the channel and manage concurrent transmissions. However, well-known MAC protocols are not suitable for this type of communication due to the previously mentioned constraints [4,5]. Therefore, novel MAC protocols tailored for WNSNs are required.

Ultimately, despite the constraints imposed by the small size of NSs, this property also enables them to access areas that their microscale counterparts cannot reach. Consequently, an effective WNSN deployment protocol must account for the limited capabilities and inherent constraints of NSs. In addition, the design of a deployment protocol should also consider the specific requirements of the intended application. These requirements determine the mobility, range, and density of the WNSN. For instance, a static topology may be required for industrial applications, whereas a dynamic topology may be more suitable for healthcare applications [15]. Similarly, battlefield monitoring demands a very high density of NSs deployed over a large area, while monitoring soldiers’ performance requires a smaller deployment area with lower density [16].

Overall, an effective deployment of a WNSN must ensure full coverage of the target area, efficient energy utilization by NSs, and continuous connectivity among NSs.

In this paper, we present a discussion on the deployment of wireless nanosensor networks (WNSNs), highlighting the challenges, requirements, attributes, and current studies related to WNSN deployment. To ensure transparency and rigor, this review follows a structured narrative approach. Relevant studies were identified through multiple reputable sources, including IEEE Xplore, ScienceDirect, SpringerLink, MDPI, and Google Scholar. The search employed keyword combinations such as “wireless nanosensor networks”, “deployment”, “terahertz communication”, “energy efficiency”, “network comnnectivity”, “Area Coverage”, and “nanoscale optimization”. While the primary focus was on peer-reviewed articles published between 2013 and 2025, several earlier seminal works were also included due to their foundational contributions to electromagnetic nanosensor design. Studies were included if they addressed deployment-related aspects, such as node placement, network topology, coverage optimization, energy efficiency, or communication reliability, and excluded if they focused solely on molecular communication or theoretical modeling that do not affect the network deployment. This structured yet flexible approach ensures comprehensive coverage of both recent advancements and key foundational studies within the interdisciplinary field of electromagnetic WNSN deployment research.

The paper is organized as follows: Section 2 lists the different types of electromagnetic nanosensors, while Section 3 provides an overview of some WNSN applications. Section 4 discusses the various characteristics of WNSNs, followed by Section 5, which presents the challenges associated with WNSN design. Section 6 then describes the types and attributes relevant to WNSN deployment. Finally, Section 7 presents the conclusions of the paper.

## 2. Types of Electromagnetic Nanosensors

NSs with graphene antennas differ in their types and capabilities. They can be classified into three categories based on the property they sense [1,2]:

1. Physical NSs: They are miniature sensing devices typically constructed from nanomaterials. These materials provide high sensitivity and ultra-low power operation, enabling the detection of physical parameters such as pressure, vibration, temperature, and mechanical deformation. Emerging quantum nanosensors offer even higher precision, potentially improving network performance [17]. In electromagnetic WNSNs, physical nanosensors are particularly relevant due to their compatibility with THz-band communication and integration with graphene-based nanoantennas. Their small size and limited energy capacity directly impact deployment considerations, influencing achievable sensing density, communication range, and energy consumption [18,19].
2. Chemical NSs: They exploit nanostructured materials with high surface-to-volume ratios, such as functionalized graphene, metal oxides, or thin nanofilms, to detect chemical substances. Their high sensitivity enables detection of gases, ions, and molecular concentrations, making them suitable for environmental monitoring and industrial applications. In electromagnetic WNSNs, chemical nanosensors are typically stationary, and their performance is affected by environmental variations that influence THz propagation. Coupled with their ultra-low-power requirements, these characteristics directly shape node placement strategies, required sensor density, and energy management within deployment frameworks [20].
3. Biological NSs: They are composed of two essential components: a bioreceptor and a signal transducer. When the bioreceptor detects any change in the targeted biomolecules, the signal transducer converts this change into a measurable output [21]. They are widely used in biomedical contexts for monitoring proteins, pathogens, metabolites, or cellular activity. Their operation in highly heterogeneous biological environments introduces deployment constraints such as limited communication range, strong THz absorption, and mobility restrictions within tissues or organs. As a result, deployment strategies for electromagnetic WNSNs using biological nanosensors must consider spatial distribution inside biological media, reliable communication, and energy-efficiency to ensure accurate and continuous physiological monitoring.

Based on the type and specialty of NSs in a WNSN, a new range of applications can be designed and developed. WNSN applications are expected to offer significant advancements in various fields. Next, an overview of some WNSN applications is presented.

## 3. WNSN Applications

As mentioned before, the concept of nano-components presents the possibility of sensing, measuring, computing, data storing and reporting events in the nanoscale, making the development of a new range of unimaginable applications possible [22]. Moreover, these new applications can be of service to several vital fields, enabling many ideas and innovations to be considered. Next, we present some WNSNs applications.

### 3.1. Medical Applications

Ordinarily, WNSNs deployed inside the human body are referred to as Body Area Nanonetworks (BANNs). When biological NSs are introduced into the human body, they can be used to enhance healthcare in various areas, such as the following:

- Diagnosing diseases by detecting the presence of viruses, bacteria [13,23] and different infectious agents [24] in the human body like cancer cells [22].
- Controlling drug delivery systems [4].
- Monitoring the level of sodium, cholesterol, glucose, and other ions in the blood [2].

Regardless of the application’s mission, BANN architecture usually includes a wireless interface, like a specialized medical equipment or cellphone. This interface is used to mediate and transfer information between NSs and the healthcare provider via internet [2].

### 3.2. Military Applications

Different types of NSs can offer significant advantages to the military industry. For instance, unlike classical chemical sensors, chemical and biological NSs can detect extremely low concentrations of chemical compounds, down to one part per billion or even a single molecule [13].

Furthermore, chemical NSs are capable of accomplishing their tasks without relying on large external equipment, such as spectroscopy devices. Instead, in a WNSN where a large number of NSs are deployed, the gathered information can be transmitted to a macro device connected to the network in a very short time [2].

Physical NSs can be used to detect very small cracks and structural damages in armor or other structures exposed to attacks in an efficient manner [2].

### 3.3. Environmental Applications

One of the main applications of WNSNs in the environmental field is for combating agricultural pests and monitoring plants [25]. This application is known as precision agriculture. The concept of precision agriculture is to sense and monitor the conditions of the farming field and its plants, for any signs of drought or pests affecting the crop, at a nano scale. In cases where such signs are detected, the irrigation or pesticide levels are adjusted immediately to protect the crop. Such technology is expected to be executable by the joint use of both GPS and satellite imaging of the targeted field with the utilization of WNSNs [2]. Another environmental application is utilizing WNSNs for monitoring and controlling the level of pollution in the air [26].

### 3.4. Intelligent Food Packing Applications

WNSNs can offer many advantages for the food industry as in [27,28]. NSs, as tiny devices invisible to the naked eye, can be embedded in food containers. Then, they can be used either in controlling the external and internal conditions of the food or as an electronic bar code to monitor the containers. Furthermore, NSs can help in monitoring the food in all the manufacturing stages (production, processing, distribution, and consumption). Finally, NSs can help in reducing the time required to detect pathogen from days into hours and potentially minutes.

### 3.5. Smart Offices

NSs can be integrated into various office supplies, such as pens, paper, and other items, making the concept of smart offices a reality [6].

There is also a wide range of other potential applications for WNSNs. However, the specific requirements of each application play a crucial role in determining how the network is deployed. These requirements include the level of coverage needed (full or partial), the positioning of the NSs (static or mobile), the density and spacing of NSs, and other factors. In the following sections, the impact of different application requirements on the deployment of WNSNs is discussed.

## 4. WNSN Characteristics

### 4.1. WNSNs Architecture

As mentioned earlier, the nanoscale nature of nano-components, specifically NSs, requires the following:

- Cooperation between NSs to accomplish the application’s mission and perform complex tasks [5].
- The presence of more capable entities to coordinate communications between NSs [29].

Regardless of the application, three components are usually considered essentials in the WNSNs architecture [10]:

1. Nanosensors (NSs): These are small devices with sizes typically around 10–100 μm${\,}^{2}$ [10]. They can perform only simple tasks, such as sensing, actuation in the small area around them, or basic computation. The density of NSs deployed in a WNSN is a major factor affecting network performance [15]. NS density depends on the size of the target area and the total number of NSs deployed. In general, NSs in WNSNs are expected to be deployed at high density, meaning a large number of NSs in a small area.
2. Nanorouters (or Nanocontrollers, NCs [13]): These are devices with greater computational capabilities than NSs. When deployed, they typically manage NSs within their cluster by performing three essential tasks using short messages [16,30]:
  (a) Scheduling NSs activities;
  (b) Gathering NSs data;
  (c) Directing NSs movements in dynamic deployments.
3. Nanogateways (NGs, or Micro-gateways, MGs): These are devices that collect information from the entire WNSN and forward it to other networks or devices, such as Wi-Fi, 5G, or LTE [31].

Nonetheless, there are some research works made for WNSN, where the network topology contained NSs only or NSs with NCs; such papers will be discussed later on this paper in Section 6. Other papers deployed NSs and a NG as in [31]. In [31], they deployed a NG attached to an Unmanned Aerial Vehicle (UAV). The UAV is used to scatter NSs in the target field.

### 4.2. Differences Between Sensors and Nanosensors

Before the introduction of WNSNs, Wireless Sensor Networks (WSNs) received significant attention from researchers, with numerous studies addressing almost every aspect of these networks. Although sensors and NSs share several characteristics, such as limited computational power and energy, they also exhibit important differences. As mentioned earlier, NSs are nanoscale devices, which magnifies their limitations compared to conventional sensors. Furthermore, the graphene-based antennas in NSs introduce additional constraints on communication. Specifically, these novel antennas require NSs to operate in the Terahertz (THz) band, which suffers from high propagation loss, thereby limiting the communication range of NSs.

Table 1 lists some differences between sensors and NSs [30,32,33,34,35].

## 5. WNSN Challenges

**Figure 1:**
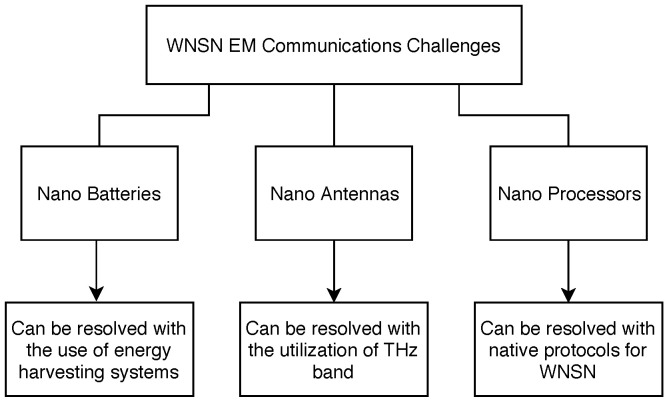
Challenges facing the development of WNSN.

Fundamentally, the small size of NSs presents the primary challenge for researchers when studying and designing WNSNs [3]. Naturally, all components in NSs are scaled down to the nanoscale, and the associated challenges can be summarized around three major aspects as illustrated in Figure 1.

### 5.1. Nano Batteries

Discussing energy harvesting systems for WNSNs is essential for several reasons:

- The limited energy that can be stored in NSs due to the small size of nano-batteries, which is typically sufficient to exchange only a few hundred bits at a time [6];
- The infeasibility of replacing or recharging the NS batteries when WNSNs are deployed with a large number of NSs over extensive geographical areas or in hostile environments [36].

Consequently, energy harvesting systems are developed to achieve the goal of guaranteeing the perpetual operation of WNSNs. However, since most WNSN applications are designed for environments, where there is no heat or light, such as inside the human body [6], studies for energy harvesting systems focus on different types of ambient energy, like mechanical energy (e.g., muscle stretching and body movement), vibrational energy (e.g., acoustic waves), fluidic energy (e.g., blood flows and body fluid), to be converted into electrical energy [22]. Such systems are enabled by the existence of nano-generators, more specifically Piezoelectric nano-generators [6]. The design of piezoelectric nano-generators has an array of zinc oxide nano wires (ZnOs) that power a commercial laser diode (LD) by converting the vibrations applied to the nano wires, at nanoscale, into electrical energy [36]. Moreover, the rate of the vibration applied to the nano wires determines the amount of energy that the nano-generator will produce. Therefore, vibration rates differ between different environments, for example, the vibration rate produced from a person tapping their foot is 1 Hz, while the vibration rate produced from a moving vehicle is 2000 Hz [6]. Hence, a stochastic model is proposed for calculating the harvested energy in NSs for different resources and locations as in [12].

Furthermore, two essential energy sources are proposed, for WNSNs, for supplying vibrations to the nano wires, in the piezoelectric nano-generator, to produce electrical energy [22]:

1. Energy from the surrounding environment: Whenever WNSN is deployed in an environment, where there is enough mechanical energy to power all NSs, exploiting such energy can represent the more convenient option for the energy harvesting system to utilize it as a source for the energy as discussed in [7].
2. Energy from the Wireless Power Transfer (WPT): WPT is an external device with ultrasound, used in piezoelectric nano-generators to vibrate and create compress–release cycles to extract energy from as presented in [22]. The usefulness of this device, as a power supply, is that it guarantees a constant energy harvesting rate regardless of the environments’ conditions as presented in [37], where a bistable piezoelectric device is proposed for activating a noise in response to the frequency available in the medium. Then, the device is designed to convert vibrations generated by the noise into electrical energy.

However, when NSs utilize these harvesting systems, their energies are constantly fluctuating. Hence, when deploying WNSN, the selected scheme for the communication between NSs has to account for this fluctuation of energy. Therefore, it has to account for both the consumption and harvesting processes jointly, to ensure the continuous connectivity between NSs [13].

### 5.2. Nano Antennas

As mentioned above, communication between NDs (either NSs or NCs) in the WNSN is a major topic. NSs are required to communicate amongst each other and with other devices to be able to transfer information and execute the network’s mission. When EM communication is utilized by the WNSN, regular optical transceivers, operating radio frequency (RF), which are known to be built-in wireless sensors, are not applicable to NDs according to [37]. Their limitations are represented in their size, complexity, and energy consumption, which led to the focus on the searching for new materials suitable for NDs and to the proposing of the graphene material [38,39]. The crucial proprieties of graphene, graphene nano-carbons (GNRs), and carbon nanotubes (CNTs) facilitated the manufacturing of nano-processors, nano-batteries, nanosensors, and nano-memories [40].

As a result, NSs’ nano-antennas are proposed to be made from graphene. To identify the appropriate frequency for this new technology, the radiation properties of the graphene must be pointed first. Studies proved that utilizing very high operating frequencies, like the Terahertz band (0.1–10.0 THz), for NSs communications is the most convenient for the electromagnetic properties of graphene antenna [5]. In addition, the Terahertz band offers many advantages to the communication scheme:

- It allows NSs to transmit and receive information in a very large bandwidth (up to hundreds of Gbps [6]), which guarantees simple communication and medium sharing mechanisms [4].
- It has a high bit rate and short transmission time, which minimizes the probability of collisions [14].

Nevertheless, the Terahertz band is a highly frequency selective band. Therefore, it is affected by the type and concentration of molecules existing in the medium, which can cause the loss of the Terahertz signal’s power and the creation of noise in the channel. Hence, different ranges of frequencies can face different sets of challenges [38].

Furthermore, the terahertz band restricts the communication range to be between only micro and millimeter distances [6]. This limitation in the communication range is caused by the combination of the high propagation loss of the Terahertz band and the limited energy of NSs [13]. Thus, determining the most convenient distance between NDs and the amount of transmission power required for NSs to guarantee a successful transmission during the deployment is mainly dependent on the range of frequencies used and the conditions of the medium.

### 5.3. Nano-Processors

The term nano-processors directly implies the limitation in NSs computational power. Therefore, NSs can perform only certain simple tasks such as computing, sensing, actuation, and storing [4]. Furthermore, this limitation demands communication between NSs as mentioned earlier. Although NSs are expected to transmit only a small amount of information in a few encoded bits [5], when all NSs are accessing the channel for communication simultaneously, MAC protocols are needed to organize this process. MAC protocols are required to execute two main tasks:

- Regulate NSs access to the channel.
- Coordinate and synchronize NSs transmissions.

However, well-known MAC protocols, like TDMA, CDMA, and CSMA/CA, are not suitable to coordinate channel access for WNSNs since they do not account for three major aspects of WNSNs [4,5]:

1. The limited computational powers of NSs, which enable the introduction of new protocols with ultra low complexity.
2. The distinctive properties of the Terahertz band such as very high propagation loss lead to limited transmission range. The propagation loss is affected directly by the distance between the communicating NSs. Therefore, a tightly synchronized access to the channel is required.
3. The fluctuation of NSs energy, caused by the utilization of energy harvesting systems as mentioned earlier.

Subsequently, new MAC protocols are required to advise for NSs communication while using the Terahertz band. Novel MAC protocols for WNSN must comply with several requirements including (1) energy efficiency of the communication scheme, (2) reliable and robust communications scheme, and (3) collision recovery schemes [5]. There are several studies that proposed different MAC protocols for the communication in WNSNs as in [5,6,13].

In conclusion, all limitations and constraints of NSs significantly impact any proposal for WNSN deployment. Therefore, the deployment of NSs must account for their limited energy and computational capabilities by either offloading complex algorithm execution to NCs or designing simple algorithms suitable for execution by NSs. In Section 6.5, we detail and discuss models that account for the effects of NSs’ constraints on deployment protocols.

Next, we present a discussion of the various aspects and challenges associated with WNSN deployment.

## 6. WNSN Deployment

In general, the term network deployment refers to the setup of the network components in a matter that will guarantee the network’s ability to fulfill the requirements of the application it is deployed for. Moreover, to obtain a good performance by the network, the deployment protocol is obligated to guarantee the following:

1. A sufficient sensing coverage for the target area (full or partial);
2. Optimize NSs energy consumption to ensure the required lifetime for the network (perpetual or temporal);
3. The continuous connectivity between NSs at all times.

For this reason, the positioning and spacing of NSs within the deployment area is a critical aspect of the deployment process. The deployment protocol must consider the medium conditions and NS energy levels to ensure that NSs can communicate effectively and report on events occurring within their coverage area. Furthermore, WNSN deployment must account for special scenarios, such as NSs switching to sleep mode to harvest energy or being destroyed by external factors. Failure to consider such situations may result in gaps in connectivity between NSs or incomplete coverage of the target area.

Therefore, to achieve the deployment protocol’s objectives of guaranteeing coverage and connectivity, different deployment attributes must be identified based on the application’s requirements:

- WNSNs can be deployed with either fixed locations for the NDs serving in the network (static deployment) as in industrial applications where NSs are used to monitor and control machines and devices remotely [41], or in areas where NSs are constantly moving (dynamic deployment), as in healthcare applications where NSs travel through the bloodstream inside the human body [15]. Later, we will present different proposals developed for both types of deployments.
- NSs Density: Some applications (e.g., applications for sensing and monitoring battlefields) require a very high density of NSs to be deployed over a large field to guarantee a satisfactory level of coverage for the target area. Other applications (like applications for monitoring soldier’s performance) require deploying NSs in a small area where fewer number of NSs can provide sufficient coverage [16].

Furthermore, in some applications, NSs are required to position themselves in the target field based on some criteria to fulfill their application’s requirements. Such deployment is named as self deployment of NSs. However, there are two major issues that could face the self deployment of NSs:

1. NDs could stack on a specific area, causing an excessive coverage for that area leading to huge waste of NDs energies, while other areas are lacking the proper coverage.
2. NDs could scatter around the environment, leading to the loss of connectivity between them. This scattering could create holes in the network’s coverage and connectivity [35].

In these two cases, the deployment of the network is considered not optimized, and a redeployment of the network must be performed. Unfortunately, although many solutions have been proposed for NSs—communications, energy harvesting systems, and connectivity between NSs—only a few papers have discussed the issue of WNSN sufficient sensing coverage. Regardless, WNSN deployment depends on the selected topology for the network and the algorithm utilized to execute the deployment. Next, we discuss the different topologies proposed for WNSNs deployment.

### 6.1. WNSN Topologies

In general, network topology defines the layout of the components’ connections inside the network. There are different shapes that the network’s topology can be: ring, star, mesh, etc. Nonetheless, the importance of the topology for the network stems from its effect on two main aspects of WNSN, according to [22].

The distance between NDs (NSs or NCs) is a critical factor in WNSN deployment, as it directly affects network efficiency. This is because distance influences absorption loss, which is the primary cause of power loss in THz signals. Moreover, absorption loss increases proportionally with the distance between communicating NDs. Hence, the distance between NDs determines the amount of transmission power required to maintain an acceptable power density at the receiver. Among several studies describing the effect of molecular absorption on Terahertz signals, Jornet et al. presented detailed models in [29,38,42].

- Selection of Communication Techniques: Since the limited energy of NSs is a major issue for WNSNs, the selection of communication protocols and schemes is a vital consideration. Therefore, the effect of the network topology on the energy consumption of the employed communication protocol must be taken into account.

Consequently, deciding on the topology in which the WNSN will be deployed is a crucial aspect of the deployment phase.

There are two main classifications proposed for the topologies of WNSNs:

#### 6.1.1. Centralized Topology

**Figure 2:**
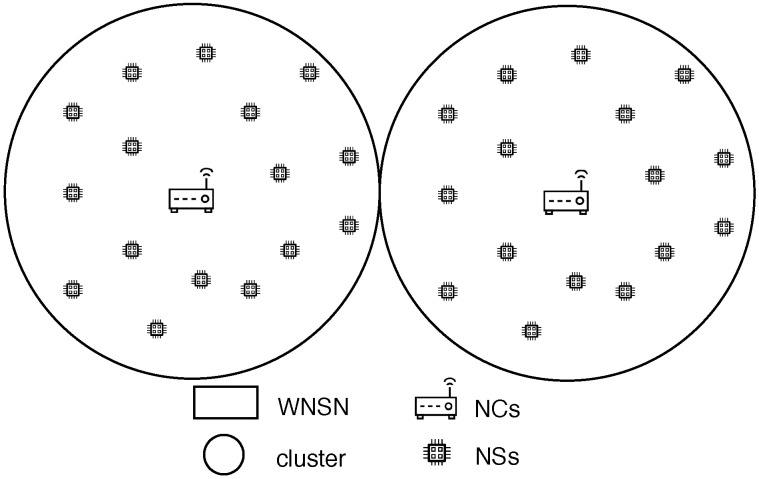
Cluster topology for WNSN.

**Figure 3:**
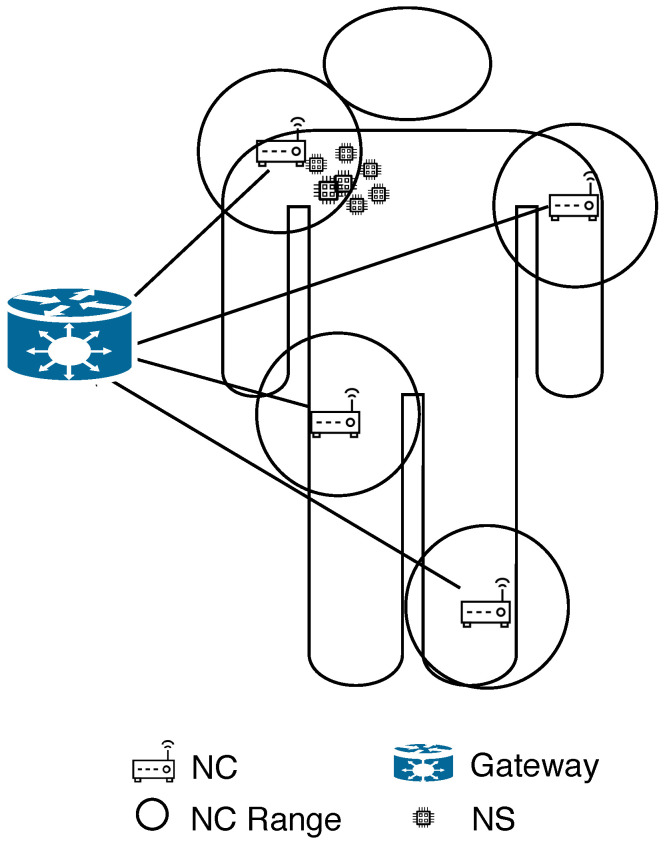
Infrastructure topology for WNSN.

WNSN has a centralized topology when NCs are stationed among NSs, to push the complexity of the network’s algorithms toward NCs. There are two models proposed for WNSNs in a centralized topology:

1. Cluster model: The cluster model, as shown in Figure 2, is where the WNSN is divided into clusters with multiple layers. Each cluster is formed based on locations. At the lowest layer of the cluster, NSs reside as the smallest nodes with the least capabilities. Meanwhile, all NSs in one cluster are managed and organized by the layer above containing NCs. NCs communicate with all NSs in their cluster through single or multiple hops. This layer helps in shifting the complexity of the protocols and algorithms from NSs into NCs, to adapt with the limited resources of NSs. At the top layer, a gateway is positioned to ensure the connection between NCs and a macro device, such as computer or a smart phone. When assigning NSs to a specific cluster, the communication between them and the center of the cluster must be guaranteed by assuring a successful arrival for the packets to the center. Different attributes affect the received Terahetz signal, carrying the information, which will be discussed in detail on Section 6.5. According to [22], while such a model is not suitable for dynamic WNSNs (where NSs are in constant moving state), it helps in reducing the energy required for data transmission, by optimizing the network address using subranges for each cluster in static WNSNs. Furthermore, cluster model facilitates the design of energy efficient communication techniques for the WNSN, where each NS can adjust its energy to adapt with different transmission parameters, such as transmission power, number of hops and so on.
  Such a model is proposed in [43] for the WNSN framework. The first step of the proposal, after deploying NSs uniformly in the target area, is to classify NSs in the WNSN into one of three cluster types: corner nodes, border nodes, and center nodes. The classification process is performed by calculating the density of each cluster type, according to some equations. Then, compare the values with the number of broadcasted packets for each NS. Thereafter accordingly, NSs are classified into a specific cluster type. Then, the second step in the proposal is to select an NS as a cluster head, rather than an NC, in each cluster type. The cluster head is chosen based on the following attributes:
  - Ratio of the NS residual energy to the NS average energy.
  - Number of neighboring NSs.
  - Number of times the NS was selected as the cluster head.
  - The distance between the cluster heads of all cluster types.
  The authors concluded that the proposed protocol reduced the energy consumed by WNSN in comparison with algorithms in which cluster heads are selected based on the residual energy only. However, the proposal did not detail any consideration for the Terahetz band peculiarities. The effect of the deployment attributes, frequency range, distance between communicating NSs and molecular composition, on the Terahertz signal has to be considered in any WNSN topology to guarantee the successful reception of the packets to their destination.
2. Infrastructure Model: The infrastructure model is the most suitable for applications in which the WNSN exhibits constant movement (mobile WNSNs), such as networks operating within the bloodstream. In this model, NCs are pre-stationed at fixed locations. As NSs move, they are not linked to a specific NC; instead, they transmit information to any NC within their current coverage range. All NCs then forward the collected data to the gateway, resulting in the highest energy consumption occurring at the NCs. However, since NCs possess greater energy storage capacity, this is considered advantageous. Moreover, due to their fixed positions, NCs can exploit external energy sources for continuous power supply. Meanwhile, NSs are activated only after harvesting sufficient energy from the surrounding medium to transmit information to the nearest NC. Nevertheless, a key concern in this model is the large number of NSs, each requiring a unique address, which significantly increases the number of bits needed for addressing. The primary application associated with this topology is Body Area Nanonetworks (BANNs). Figure 3 illustrates an example of WNSN operating under an infrastructure topology. However, the transmission power of NSs is constrained by the technology used to manufacture their antennas [13]. As a result, the transmission range—within which packets can be successfully received by another ND—is very limited. Therefore, when deploying a WNSN in an infrastructure topology, the placement of NCs must account for the various attributes affecting the Terahertz signal to ensure that any moving NS can successfully transmit its packets to at least one NC. The attributes influencing Terahertz signal reception are discussed in Section 6.5.

#### 6.1.2. Distributed Topology

**Figure 4:**
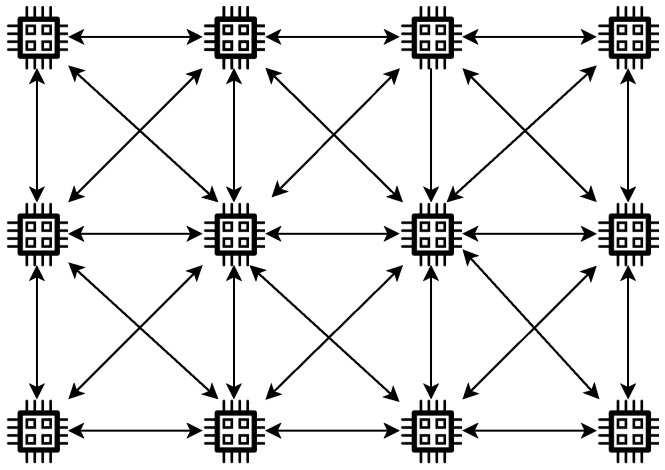
Mesh topology for WNSN.

In distributed topology, NSs are required to handle the mission of the application and to communicate with the micro world on their own. A model is proposed for such a topology called the mesh model. The mesh model is suitable for WNSNs designed to monitor part of an area using a fixed number of NSs in the medium [44]. An example of such a network is WNSNs composed of identical NSs deployed in metamaterial called SDM (software-defined metamaterials). SDMs are materials with reconfigurable electromagnetic properties. Their properties can be altered by some software using the NSs existing in their structure [45]. The main advantage of such a model is that there is only one type of NS in the WNSN, and they communicate using flood-based schemes. In the mesh model, when any NS receives a packet, it is required to check the destination address in the packet. In case the address does not match its own, the NS is obligated to retransmit the packet to its neighbors. As a result, any two NSs, not in each other’s transmission range, are guaranteed to always have a multi-hops path connecting them. To avoid the isolation of any NS in such a model, the NSs have to be positioned in the target area based on the medium conditions, in which for any NS, there is at least one NS that can receive the packets successfully. However, to implement such a model, NSs are required to always listen to the channel causing most of the energy harvested to be exhausted for retransmitting other NS packets rather than their own useful information. Figure 4 illustrates the concept of mesh topology.

In conclusion, both distributed and centralized topologies have their respective advantages and limitations. Therefore, selecting the most appropriate option depends on the specific application, its requirements, and the network’s characteristics. Moreover, the placement of NSs in any topology must consider the deployment attributes that influence the successful transmission of the Terahertz signal, including the frequency range, the distance between communicating NSs, and the molecular composition of the communication medium.

Next, a discussion of the different types of WNSN deployments from multiple perspectives is presented.

### 6.2. WNSN Deployment Types

The goal of the deployment protocol is to produce an optimized deployment for WNSN. An optimized deployment refers to the full coverage of the target area and the continuous connectivity between NDs with the least number of NDs possible. To achieve that, the deployment of WNSN must account for the different methods for the positioning of NSs in the target area, NSs mobility, and the deployment dimension (2D or 3D) as follows.

#### 6.2.1. The Deployment of Static NSs

As mentioned above, in the static deployment of WNSNs, NSs are positioned at fixed locations in the deployment field. This positioning is usually decided upon based on the application’s requirements (full or partial coverage, perpetual lifetime for the network or temporal, etc.), the attributes of the communication channel (frequency, path loss, noise, etc.), and NSs energy consumption (transmission power, energy harvesting rate, etc.). When all of these attributes are considered, both the WNSN continuous connectivity and full coverage of the target area are guaranteed. Nonetheless, when the deployment of the WNSN starts with a prefixed location, NSs will remain static in their locations, which makes such deployment suitable for applications designed to monitor static environments, such as battlefields or agricultural fields. Furthermore, static deployment can be used for either a two-dimensional (2D) area or three-dimensional (3D) area.

**Figure 5:**
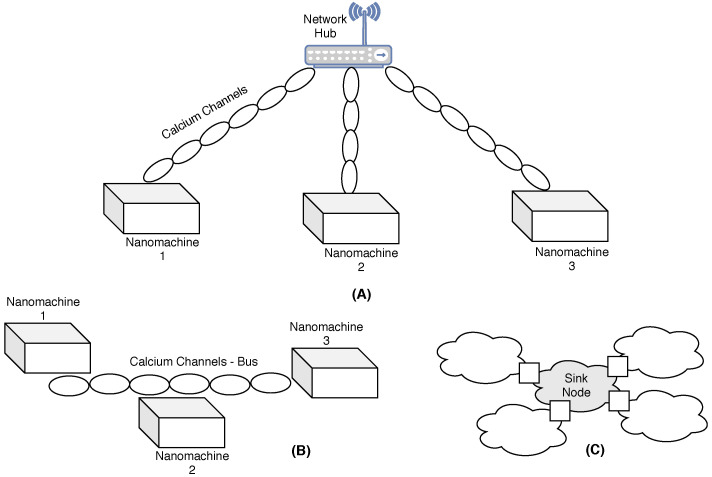
Different static deployment strategies: (**A**) Star topology. (**B**) Bus Topology. (**C**) Grid Topology.

In two-dimensional (2D) deployments, all NSs are positioned at the same height but at different planar coordinates (x, y). In [46], three distinct static deployment strategies are proposed for a 2D area, utilizing molecular communication between NDs as illustrated in Figure 5. These three strategies employ calcium signaling to support three different topologies as follows:

1. Star topology: All NSs are connected to a central device via dedicated calcium signaling channels. The central device is responsible for facilitating the communication between any two NSs.
2. Bus topology: All NSs are connected via a bus of calcium cells. Any NS can send data to another NS by triggering the calcium cell closest to it to send a signal representing the data. The signal keeps propagating through the bus of cells until it reaches the closest cell to the receiver. Then, the receiver cell will decode the information and send it to the receiving NS using another molecular communication scheme.
3. A grid topology: Only a sink node is deployed and calcium cells are used as NSs, exploiting the signaling capability of calcium cells. The grid is formed by calcium cells division, around the sink, until it reaches a certain distance as required.

All three proposed deployments are described as scenarios only and they focus on the use of calcium signals inside the human body.

In three-dimensional (3D) deployments, NSs are positioned according to three coordinates (x, y, z), resulting in their placement at different heights. A 3D model for static WNSNs is presented in [47], where the authors propose a framework for calculating path loss in the Terahertz channel for a WNSN deployed on a plant using a cluster topology. The model assists in determining the number of NSs required to achieve adequate area coverage. The proposal includes a structural model of the plant that considers various attributes such as plant size, number of leaves, and moisture distribution. The plant is represented as a series of concentric cylinders, with the center aligned along the stem. The model also accounts for variations in leaf types among different plant species. Furthermore, it is divided both vertically and horizontally: the horizontal divisions represent regions with different moisture levels, while the vertical divisions correspond to different parts of the plant. Finally, a model for determining the probability of successful transmissions, utilizing the two previous models, is proposed as well.

Nonetheless, when NSs are deployed in fixed positions, the conditions of the medium must be considered, and adjustments to their positions should be made if those conditions change. For instance, when the water vapor concentration in the communication medium increases, the distance between NSs must be reduced to ensure the successful reception of the Terahertz signal while maintaining the same frequency range.

However, certain environments in which NSs are deployed—such as intrabody environments—are unsuitable for static deployment. In such cases, the dynamic deployment of NSs represents a more viable option, which is discussed next.

#### 6.2.2. The Deployment of Dynamic NSs

As mentioned earlier, the small size of NSs enables them to explore otherwise inaccessible locations, allowing the development of new types of applications. For such applications, NSs must be deployed with mobility in mind, following a dynamic deployment approach [10], and more specifically, a self-deployment strategy.

In self-deployment, NSs are initially positioned randomly and then execute predefined instructions that guide them in identifying optimal positions to relocate to, ensuring full coverage of the target area and continuous network connectivity [35]. This approach is referred to as self-deployment because NSs determine their new positions autonomously based on specific optimization criteria.

Depending on the environment, once NSs reach their optimal positions—where the application’s objectives are satisfied—they may either continue to move (as in the case of NSs deployed in the bloodstream) or remain stationary (as in battlefield monitoring applications) until environmental changes occur that necessitate redeployment.

**Figure 6:**
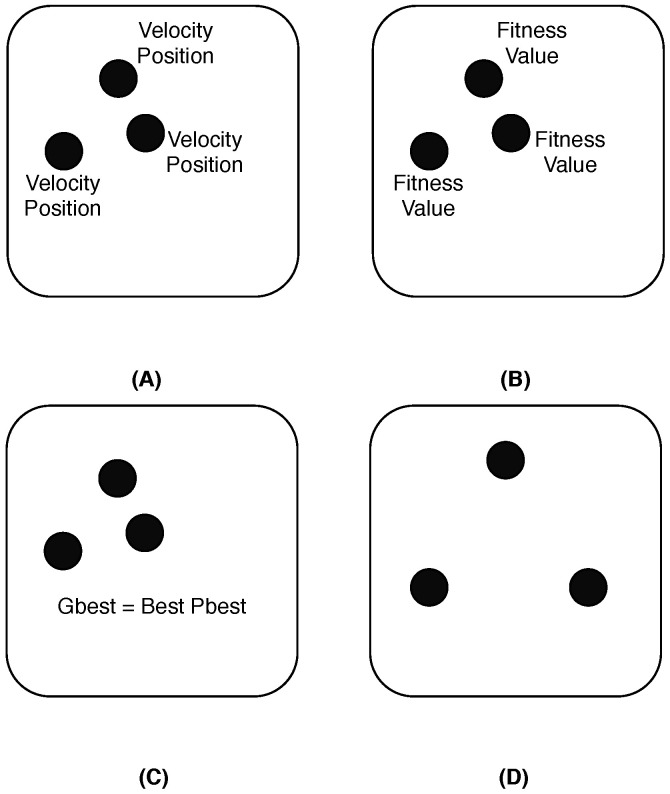
Steps for executing PSO: (**A**) Initialize position and velocity for all particles. (**B**) Calculate Fitness Value and compare with Pbest. (**C**) Compare Pbest for all particles. (**D**) Update all positions and velocity vectors.

One of the algorithms used to propose for WNSN self deployment, called the Particle Swarm Optimization (PSO) algorithm in [30,33]. The PSO algorithm is a set of potential solutions for a moving swarm of particles using the principle of self-organizing. PSO relies on a fitness function which aims to find the optimal distribution of particles in the environment based on different attributes. It pushes each particle to find its best position (Pbest) while considering the global best position (Gbest) of the whole swarm, to fulfill the goal of the fitness function [48]. In the context of WNSN deployment, the Particle Swarm Optimization (PSO) algorithm operates as an iterative optimization mechanism, in which each nanosensor (particle) adjusts its position within the target area based on both its own experience and the collective behavior of the swarm. At each iteration, nanosensors evaluate their fitness—typically expressed in terms of the achieved coverage ratio or connectivity level—and update their velocity and position toward the best solutions identified locally and globally. Through this continuous adaptation, the network gradually converges toward an optimized configuration that maximizes sensing coverage while maintaining connectivity between nanosensors. The PSO approach has been widely recognized for its simplicity and effectiveness in self-organizing mobile nanosensors; however, it remains computationally demanding for devices with highly constrained energy and processing capabilities. Therefore, most of the current research focuses on lightweight adaptations of PSO suitable for nanoscale implementations. Figure 6 illustrates the steps for executing PSO as follows:

- Initialize the current positions and velocities for all particles in the field. Then, set the first position as the best position for each particle.
- All particles start executing the fitness function. Then, each particle starts comparing the result to their own best position. If the fitness function result is better, then change the best position to the new value obtained from the fitness function.
- Compare the best positions of all particles and set the best position as the global best position.
- Update all velocities and positions for all particles based on the global best position and move the particles to the new positions.

Many papers have proposed different deployment algorithms for WSN based on PSO as in [48]. While these proposals proved their effectiveness in area coverage and connectivity compared with the native PSO, they cannot be applied directly to WNSN due to the constraints in NSs and Terahertz band properties. Hence, some researches have proposed 2D deployment algorithms for mobile NSs based on PSO after adapting it to the WNSN constraints. The first proposal was made by Hla et al. in [49]. The proposal targeted nanorobots, assumed to be made by NSs, moving inside a human body. The authors proposed an algorithm, used as a fitness function for PSO, considering the whole network’s coverage as a criteria to judge the calculated positions. In simulation, NSs flow in the blood until they find their target. Then, they start to send signals attracting others to cover the area and form a WNSN. NSs communicate by updating their velocities based on best global position and the best local position of each NS. They keep moving and evaluating different positions until a full coverage of the area is achieved. While the proposal proved its effectiveness in the self-organizing of NSs mobility, it dismisses the energy consumption aspect in the WNSN.

Another proposal for organizing NSs mobility based on PSO was introduced in [30]. It aimed to improve the WNSN coverage and lifetime. The authors proposed a distributed deployment scheme, utilizing the PSO algorithm, for WNSNs to ensure good coverage of the target area. NSs perform the PSO algorithm with the goal of guaranteeing full coverage as the utility function. Therefore, they keep moving and changing their positions until the network reaches a state where it is sufficiently k-covered, meaning every point in the field is covered by at least k number of NSs, where k is a predefined constant. However, PSO is still a complex algorithm, and its suitability for the limited computational power of NSs is considered unclear.

Another proposal for managing NSs mobility is introduced in [50]. The proposal exploited the fuzzy logic to control NSs movements on the field. Fuzzy logic is a type of reasoning resembles the human logic. It is created to handle partial truth represented as variables, rated with numbers between 0 and 1 (where 0 is completely false, and 1 is completely true). The WNSN has NCs stationed statically and deployed in predefined positions, while NSs are deployed randomly and move constantly with a fixed velocity. NSs transmit their packets to the best NCs based on the fuzzy logic scheme. The fuzzy logic scheme utilizes the distance between NS and NC, traffic load, and residual energy of the NC as the criteria to decide on the best NC, using fuzzy numbers and fuzzy membership function. Then, the best NC will assign the NS a time slot to send its data to the NC. All NCs are responsible for communicating the information to the micro device connected to the network.

In [51], a 3D model for mobile WNSN is proposed. NSs are deployed with a 3D hexagonal cell shape according to the homogeneous spatial Poisson process deployed to sense organs inside the human body. The targeted area is described as cylindrical shape 3D hexagonal pole representing the organs, in which for any hexagonal cell, there is only one active NS. NC is deployed in the center of the cylindrical shape and acts as an active NS for that cell. The model is evaluated based on the energy efficiency when using multiple transmission methods (single, multiple, and hybrid) and aims to conclude the ideal attributes to model WNSN deployed for the organs inside the human body.

Recent research has explored the use of Artificial Intelligence (AI) and machine learning (ML) to enhance WNSN deployment strategies. These intelligent methods aim to dynamically adapt node placement, communication parameters, and energy management according to environmental conditions and network feedback.

In [52], the author investigated the use of machine learning to optimize nano-router localization in wireless nanosensor networks (WNSNs). The proposed algorithm aims to predict the number of required nano-routers depending on the network size for the maximum node coverage in order to ensure direct data transmission by estimating the best location of these nano-routers. By predicting optimal router positions based on network topology and node constraints, the approach improves connectivity, reduces communication delays, and enhances energy efficiency. This AI-driven strategy demonstrates that intelligent deployment can mitigate some of the key challenges in WNSNs, including limited communication range, energy constraints, and reliability in dense nanoscale networks. Incorporating such techniques can guide future work toward adaptive and self-optimizing nanosensor deployments.

Table 2 classifies some papers based on topologies, NSs mobility, and deployment environments.

In any case, as previously mentioned, an effective deployment must ensure sufficient coverage of the target area, energy efficiency of the WNSN, and continuous connectivity among NSs. The following sections present discussions on WNSN sensing coverage and NS connectivity.

### 6.3. Connectivity Between Nanosensors

Whether a WNSN deployment is centralized (where an NC is responsible for positioning NSs in the target area) or distributed (where each NS is responsible for positioning itself in the target area based on specific criteria), static (i.e., NSs are deployed in fixed positions), or mobile (i.e., NSs are in constant motion within the target area), the deployment must ensure that there is always a path connecting any NS to the NC (in a centralized topology) or to any other NS (in a distributed topology). Loss of connectivity between NSs could lead to a partitioned network, where information from a portion of the target area is not reported back to the micro world.

For centralized deployment, NCs are always aware of the locations of all NSs in their cluster. Therefore, NCs can determine the transmission power each NS needs to maintain constant connectivity with others. Furthermore, if an NS becomes inactive for any reason, the NC in that cluster is expected to compensate by replacing it with another active NS. As a result, connectivity between the NC and all NSs is largely guaranteed at all times as studied in [55]. In [55], the authors proposed a channel-aware forwarding scheme for WNSNs, adapting typical NS schemes to account for WNSN-specific constraints. They deployed WNSNs in a centralized manner under the following assumptions: first, the sink node is aware of the locations of all NSs; second, the sink node is responsible for controlling and managing NSs, including determining the most efficient paths between all NSs and the sink; third, all NSs have sufficient energy to transmit data. Finally, there are no channel collisions, eliminating the need for packet retransmissions.

On the other hand, in distributed deployments, since there is no central entity that has the information about all NSs, the responsibility for ensuring the continuous connectivity between all NSs lays with the NSs. For that, the transmission power used by all NSs must adapt to the distance between them. Furthermore, the routing scheme must ensure that there is always a path between all NSs in the WNSN [58]. Determining the amount of transmission power depends on the channel model, which will be discussed later. There are different approaches to ensure packet arrivals for all NSs in distributed deployments such as

- Using flood-based schemes for transmitting packets: unfortunately, it usually causes a broadcast storm due to the high density of NSs in WNSNs which leads to the problem of redundancy and collision.
- Utilizing the dynamic infrastructure approach (DIF) [53,56]: It is proposed to reduce packet retransmission rate without affecting the network connectivity. The main idea of the DIF is that NSs, with good-quality reception, are the only nodes allowed to retransmit packets; they are called infrastructure NSs. Other NSs that operate on receiving mode only are called user NSs. The process of classifying the mode for each NS to operate on is run by each NS locally (called the Maturity Process). The process is based on the NS packet reception statistics. As a result, the NS will decide whether it is better to mature to infrastructure and take the role of retransmission, or to stay as a user. Even though the DIF provides a better energy-efficient scheme than the first approach, it still suffers from flooding the network with retransmitted packets even when it is not necessary.
- Clustering-based hop-counting mechanism is presented in [54]. In this mechanism, NSs are grouped into clusters, and only the cluster leaders are allowed to communicate with one another. The main objective of the mechanism is to reduce the number of retransmissions while ensuring a high packet arrival rate.
- An efficient data dissemination scheme was proposed in [59] for an application inside the human body. In the proposal, multiple mobile NSs are deployed inside the human body traveling with the bloodstream. Furthermore, multiple NCs and 1 NG are deployed in prefixed positions, where the NG is stationed at the middle of the artery and it is the destination for all NS packets. The proposal aims to reduce the amount of energy consumed by the WNSN while maintaining the packet transmissions ratio. For that, the scheme starts with all NCs broadcasting their locations to all NSs in the WNSN. Next, each NS will start identifying the closest NC to it, based on the distance separating them. When a NS is ready to transmit packets to the NG, it first checks whether the NG is within its transmission range. If so, the NS transmits the packets directly. If not, it checks whether the nearest NC is within range and, if available, transmits the packets to the NC, which then forwards them to the NG. Otherwise, the NS sends its packets to the nearest neighboring NS that is closer to the NC. Compared to the flooding approach, the proposed scheme has been shown to reduce energy consumption while maintaining a high packet transmission rate.

Regardless of the WNSN deployment attributes, ensuring connectivity between NSs means that the packets from any NS successfully reach their intended destination. A packet is considered successfully received if the signal carrying it is readable by the receiving NS, which occurs when its power exceeds a target Signal-to-Noise Ratio (SNR). The received signal power is influenced by multiple factors: the distance between the communicating NSs, the communication frequency, and the transmission power. Furthermore, because WNSNs operate in the Terahertz band, molecules present in the medium significantly affect the received signal power. Therefore, any WNSN deployment proposal must account for the constraints of Terahertz communication to ensure successful packet reception, thereby maintaining continuous connectivity among NSs. In Section 6.5, we provide detailed models for calculating the received signal power.

Ultimately, ensuring the continuous connectivity between all NSs (and NCs if they exist in the network) is a major aspect to propose good deployment for WNSN.

Next, a discussion about the different attributes of WNSN coverage of the target area is presented.

### 6.4. WNSN Coverage

**Figure 7:**
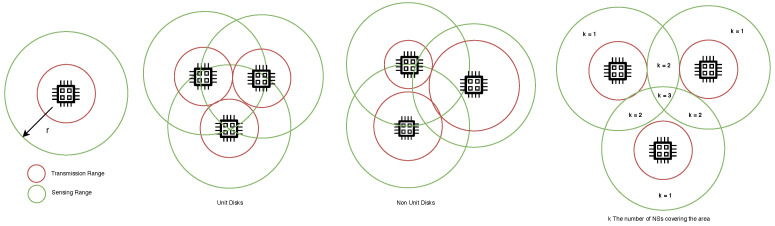
Coverage represented by a disk with unit radius (r).

When NSs are deployed in an area, they are expected to monitor and report events occurring in the entire target area. The coverage of the area can vary between covering the entire area where the WNSN is deployed (100% coverage), or part of the area (less than 100% coverage), depending on the applications requirements. However, in this paper we use the term target area in general for either partial or full coverage. Therefore, when deployed, NSs have to be located in a matter that will guarantee that any point in the area is covered by at least one active NS (in this case, the coverage is called 1-covered area). In some cases, each area is required to be covered by more than one active NSs, i.e., k-covered area, as illustrated by Figure 7, where k is a predefined constant, in the deployment algorithm, depending on the applications requirements. In [60], a proposal for deploying Energy Harvesting Cooperative Wireless Sensor Networks (EHC-WSNs) is introduced. EHC-WSNs are a new type of SN, where both energy harvesting and wireless power transfer techniques are utilized. In the proposal, two types of nodes are deployed: cooperators and receivers. Cooperators are nodes deployed for the goal of harvesting energy from the surrounding area and transferring energy wirelessly to the receiver nodes. Receivers are nodes deployed to sense the target area. A receiver node is positioned in the targeted area, in which it is 3-energy-covered by cooperators in two layers. The goal of the coverage is to satisfy the energy and sensing requirements, which is fulfilled by the 3-coverage strategy.

Nonetheless, the NSs’ coverage is represented by their sensing range. Hence, the area located within the NSs sensing range is considered covered by that NSs. Therefore, in a 2D area, NSs in the network can be identified as disks, with unit radius r, and they are connected to all NSs intersected with their disks. The NSs’ disks can either be as follows:

- A unit disk, when all NSs have the same sensing range [33,61].
- Non-unit disk, when the sensing range of NSs differs [61].

Figure 7 shows the difference between unit and non-unit disks. Such classification can help in investigating NSs coverage of the area, as in [33], where NSs are represented as unit disks. Each NS is located in 2D coordinate (x,y) with a sensing range r, and each point in the area is covered by at least k NSs, where k is a predefined number.

To ensure adequate coverage of the targeted sensing area in a specific deployment, a sufficient number of NSs must be deployed in the network. Proper placement of NSs helps achieve the desired coverage of the target area while maintaining continuous connectivity between NSs. Previous studies have identified some regular geometric patterns for sensor placement that can achieve full coverage and connectivity in both 2D and 3D environments using the minimum number of sensors, such as the following:

- In 2D environments [62]: It has been proved that positioning sensors in a triangular lattice, as shown in Figure 2, will result in an optimal deployment when the following conditions are met:
  - There is a fixed distance between NSs ($D_{th}$).
  - Sensors have a sensing range (r).
  - Each sensor is located at a triangle vertex and has 6 neighboring sensors.
  - The full coverage is achieved when the distance between the sensors is calculated as
    (1) $$D_{th}=\sqrt{3}r.$$
  - Consequently the full connectivity is achieved when the transmission range ($T_{r}$) is defined as
    (2) $$T_{r}\geq \sqrt{3}r.$$
- In 3D environments [63]: The optimal deployment can be obtained by a truncated octahedron placement for the sensors, where each sensor has 14 neighboring sensors. The truncated octahedron has 14 faces (8 faces are regular hexagons, and 6 faces are squares). In such a placement, to achieve optimal deployment, the distance between sensors can be defined based on the type of the face they are in, as follows:
  - In a square face:
    (3) $$D_{th}=\frac{4r}{\sqrt{5}}.$$
  - In a hexagonal face:
    (4) $$D_{th}=\frac{2r\sqrt{3}}{\sqrt{5}}.$$

Nonetheless, as mentioned earlier, various properties of NSs, including their sensing capabilities, are still under investigation. Several studies have proposed different techniques to enhance the sensing abilities of NSs for a variety of applications, such as those described in [64,65,66,67].

However, when NSs communicate using the Terahertz channel, the distance between them significantly affects the signal. In other words, as the distance between communicating NSs increases, both the noise power and channel attenuation also increase, resulting in a weaker Terahertz signal. Consequently, higher transmission power is required to maintain connectivity and coverage between NSs. Nonetheless, determining the appropriate transmission power must also account for the NSs’ limited energy and capabilities. To address this, the following section presents models of the Terahertz channel—including attenuation, noise, and capacity—to guide the selection of the most suitable distance between NSs in a WNSN.

### 6.5. Terahertz Channel Models

When operating in the Terahertz band for communication between NSs, molecules in the channel are the primary source of absorption and noise for the propagating signal. This phenomenon significantly influences the choice of an appropriate deployment distance between NSs to ensure effective network performance. However, the absorption caused by gas molecules varies depending on the type of molecule. Moreover, it differs between isotopologues of the same gas. Therefore, to accurately estimate the impact of molecular absorption, the absorption coefficient k must be calculated for each isotopologue of each gas present in the communication medium. Studies have shown that water vapor molecules cause the most significant attenuation and noise in the Terahertz signal [13].

Additionally, the noise power and the loss of Terahertz signal power are the two most critical factors influencing the choice of distance between NSs. These factors ultimately determine both the required transmission power and the achievable data rate for each NS.

A well-designed WNSN deployment must consider NSs’ energy constraints alongside the medium’s conditions to ensure sufficient coverage and continuous connectivity. Therefore, evaluating the impact of any proposed distance between NSs is a crucial step in designing an effective deployment protocol for WNSNs.

Next, we present models for calculating the various factors affecting the Terahertz signal, taking into account the unique characteristics of the Terahertz band.

#### 6.5.1. Terahertz Signal Attenuation

There are two main sources of Terahertz signal attenuation: the absorption of the signal by molecules in the medium, $A_{m}$, and the standard spreading loss of any propagating signal, $A_{s}$. They are as follows:

(5) $$A_{\mathrm{Total}}\left(f,d\right)=A_{m}\left(f,d\right)\times A_{s}\left(f,d\right).$$

where *f* is the frequency and *d* represents the distance between the communicating NSs.

Molecular Absorption Loss:

It is a well-known fact that each type of molecule, in general, has its own resonant frequency. When molecules are exposed to this frequency, they will start absorbing them immediately, causing the molecules to vibrate. Therefore, when the Terahertz signal travels through the medium (air, blood, oil, etc.), it could approach this resonant frequency, causing the molecules to start absorbing some of the signal’s energy and converting it into a kinetic one, which leads to the attenuation of the signal’s energy. This unique feature is associated with the Terahertz band and can be captured with the following equation:

(6) $$A_{m}\left(f,d\right)=\frac{1}{\tau (f,d)}.$$

where A${\,}_{m}$ represents the molecular absorption of the signal’s power, *f* is the utilized frequency, *d* represents the distance between the communicating NSs, and τ represents the medium transmittance which counts for the effect of the molecular absorption on the signal. The medium transmittance (τ) describes the amount of the EM radiation that is able to pass through the medium at a given frequency. It can be calculated as

(7) $$\tau \left(f,d\right)=e^{-k(f)d}=e^{-\sum k_{GI}\left(f\right)d}.$$

where *k* represents the absorption coefficient of the molecules of gas *G* with isotopologue *I*. The overall absorption coefficient for one gas is defined as the summation of all isotopologues coefficients of that gas.

However, different types of molecules have different absorption rates, based on the molecules’ physical properties (spatial orientation, bond structure, etc.) which can be measured by the absorption coefficients [29]. The calculation of the absorption coefficient can be obtained from the HITRAN database based on the model presented in [38].

Spreading Loss:

When any signal travels through any medium, it usually suffers from the loss of its power with distance. Such loss ($A_{s}$) can be described as

(8) $$A_{s}\left(f,d\right)={\left(\frac{4\times \pi \times f\times d}{c_{0}}\right)}^{2}.$$

where *f* represents the utilized frequency, *d* represents the distance between the communicating NSs, and $c_{0}$ is the speed of light in the targeted area (which is approximately 3 × 10${\,}^{8}$ m/s in the open air).

By observing Equation (8), it is noticeable that the spreading loss of the Terahertz signal can be large for long distances between NSs. As a result, the transmission range applicable for NSs can be affected, causing some inconveniences for WNSN applications [38].

#### 6.5.2. Noise in Terahertz Channel

When molecules are exposed to their resonant frequencies, they are not just absorbing part of the signal’s energy but they could also be a source for the noise in the medium as well. When the molecules start vibrating internally, which is provoked by the signal as mentioned earlier, an EM radiation will be released in the medium at the same frequency of the signal, causing the presence of noise power. In [38] a model to compute such a phenomenon is introduced using the emissivity of the channel (ε) as follows:

(9) ε(f,d)=1−τ(f,d)

where ε is the emissivity of the channel, *f* represents the utilized frequency, *d* represents the distance between the communicating NSs, and τ represents the medium transmittance as introduced in Equation (7).

Moreover, to calculate the equivalent noise temperature ($T_{\mathrm{mol}}$), detected by the graphene antennas, the authors in [38] proposed the following equation:

(10) $$T_{\mathrm{mol}}\left(f,d\right)=T_{0}\varepsilon \left(f,d\right)$$

where *f* represents the utilized frequency, *d* represents the distance between the communicating NSs, ε is the emissivity of the channel as introduced in (9), and $T_{0}$ represents the temperature of the medium.

Finally, to calculate the power of the molecular absorption noise, the applied bandwidth must be defined (which will be discussed later on). Then, the noise power $\left(P_{N}\right)$ can be calculated as

(11) $$P_{N}\left(f,d\right)=B_{3db}\left(f,d\right)\times K_{B}\times T_{\mathrm{mol}}$$

where *f* represents the utilized frequency, *d* represents the distance between the communicating NSs, $K_{B}$ is the Boltzmann constant ($K_{B}=1.3806\times 10^{-23}$ J/K), and $T_{mol}$ represents the noise temperature.

**Figure 8:**
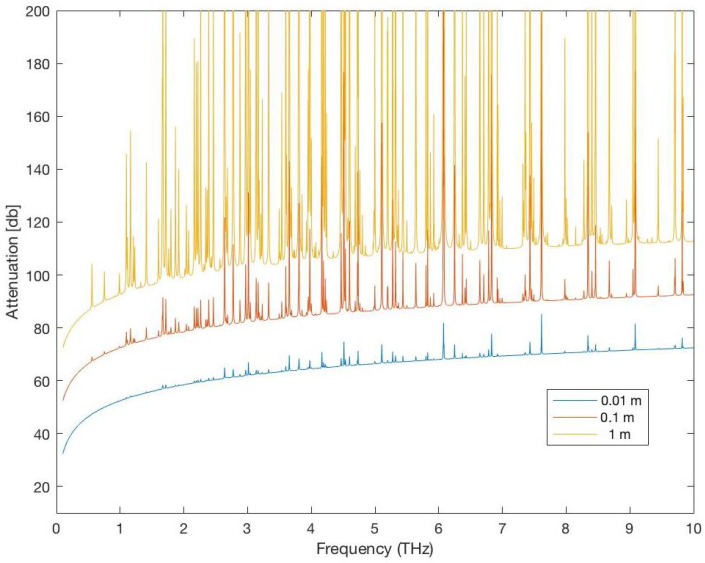
Total attenuation of the Terahertz signal in a medium with 1% water vapor.

**Figure 9:**
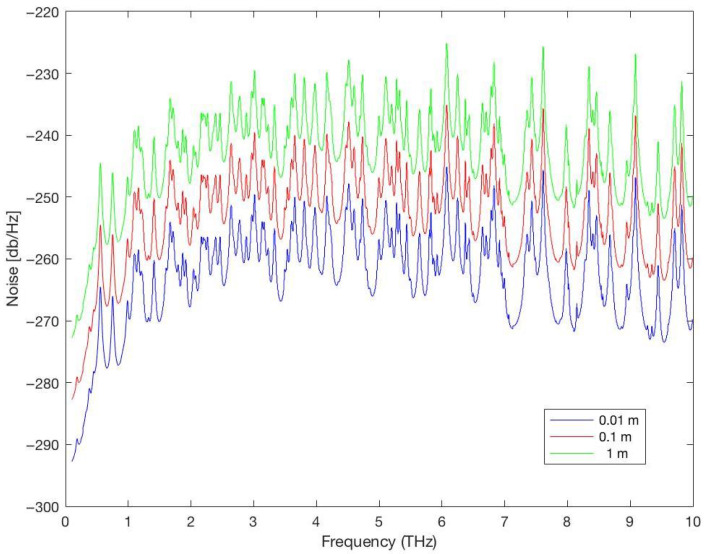
Noise in a medium with 1% water vapor.

Figure 8 and Figure 9 show the total attenuation affecting the Terahertz signal (molecular absorption loss and spreading loss) and the noise affecting the Terahertz signal for distances = 0.01, 0.1, 1 m in a medium with 1% water vapor. As expected, the attenuation and noise increase when the distance between the communicating NSs increases. Therefore, the choice of the distance between NSs is a very important choice in the WNSN deployment and affect all aspects of the WNSN performance.

#### 6.5.3. Therahertz Channel Bandwidth

Generally, the bandwidth of the Terahertz channel depends on the distance between the communicating NSs and the composition of molecules in the medium [29]. However, 3 db bandwidth (B3db) is a function of the range of frequencies that depends on the distance between the NSs [13] as follows:

(12) $$B_{3db}\left(d\right)=\left\{f|A_{\mathrm{Total}}\left(f,d\right)P_{N}\left(f,d\right)\leq 2A_{\mathrm{Total}}\left(f_{0}\left(d\right),d\right)P_{N}\left(f_{0}\left(d\right),d\right)\right\}$$

where *f* is the utilized frequency, *d* represents the distance between the communicating NSs, and $f_{0}$ is the center frequency.

However, as we discussed earlier, to ensure the NSs connectivity, the deployment of WNSN must position NSs in the target area in distances that will guarantee the successful reception of the NS packets. Next, we discuss the calculations for the signal’ transmission power and the received signal power to further explain how they affect WNSN deployment.

#### 6.5.4. Terahertz Signal Total Transmission Power

To guarantee a continuous connectivity between NSs, the value of the transmission power used by NSs has to be utilized to guarantee a target SNR at the receiver. In order for that to be true, NSs must acquire the ability to have dynamic control of the transmission power [13]. In such a case, the transmission power can be calculated as follows:

(13) $$P\left(d\right)=SNR\int_{B_{3db}}A_{\mathrm{TOTAL}}\left(f,d\right)P_{N}\left(f,d\right)df.$$

where SNR is the constant target Signal-to-Noise Ratio, and $B_{3db}$ is the channel bandwidth.

**Figure 10:**
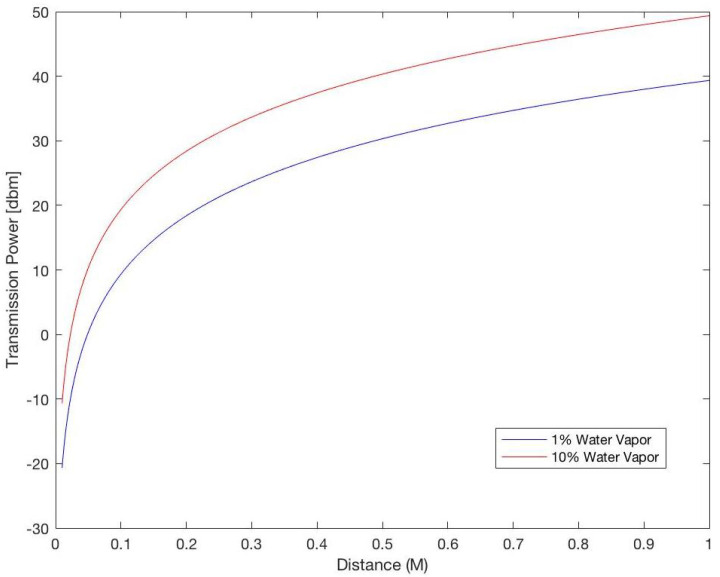
Transmission power required to guarantee a constant SNR.

Figure 10 shows the values of the required transmission power for distances between 0.01 and 1 m for different water vapor percentages in the medium, and proves that increasing the distance between the communicating NSs will increase the required transmission power to guarantee the correct reception of the information.

#### 6.5.5. Terahertz ${\mathrm{Signal}}_{p.s.d}$

To distribute the total power of the signal over the used frequencies, three different power allocation techniques are proposed and analyzed in [29]. The first is a straightforward option by distributing the power evenly throughout the entire Terahertz band as follows:

(14) $$S_{p.s.d}\left(f\right)=S_{\mathrm{even}}\;\mathrm{for}\;\mathrm{all}\;f\in B\;,0\;\mathrm{otherwise}$$

For the second option, the signal p.s.d is used to maximize the channel capacity by restricting it to the water filling principle. Such an option is possible when assuming that the total power of the transmitted signal is finite. In such a case, the ${\mathrm{signal}}_{p.s.d}$ could be defined as follows:

(15) $$\begin{array}{c} S_{\mathrm{optimal}}\left(f\right)+A_{\mathrm{Total}}\left(f,d\right)P_{N}\left(f,d\right)=K\;\mathrm{and}\\ S_{\mathrm{optimal}}=0\;\mathrm{if}\;K<A_{\mathrm{Total}}\left(f,d\right)\times P_{N}\left(f,d\right) \end{array}$$

where *K* is a constant depending on the total transmitted power of the signal, in which they introduce it as a design parameter.

However, despite the simplicity of the previous techniques, the feasibility of them is questioned due to NSs limited capabilities.

Hence, a third allocation technique is proposed based on the new advancements in graphene antennas, allowing the use of short pulses with a 100 femtosecond duration to be used as a communication technique between NSs. Researches proved that using short pulses, 100 femtoseconds long, for communication between NSs within the distance of 1 m is the best option for WNSNs [12,29,38]. Such pulses require energy that can be contained in the Terahertz band. The ultra short pulse is described in Gaussian shape:

(16) $$p\left(t\right)=\frac{a_{0}}{\sqrt{2\pi }\sigma }e^{-\frac{{\left(t-\mu \right)}^{2}}{\left(2\sigma^{2}\right)}}$$

where $a_{0}$ represents the normalizing constant to adjust the pulse total power, σ represents the standard deviation of the Gaussian pulse in seconds, and μ represents the location in time for the center of the pulse in seconds. While the time derivation of this Gaussian pulse can be acquired by the combination of nanoscale delay lines. Nonetheless, the time derivative p.s.d. for such a femtosecond-long pulse has also a Gaussian shape as follows:

(17) $$S_{\mathrm{pulse}}^{\left(n\right)}\left(f\right)={\left(2\pi f\right)}^{2n}a_{0}^{2}e^{-{\left(2\pi \sigma f\right)}^{2}}$$

#### 6.5.6. Terahertz Received Signal Power

When NSs send signals, the power of the signal calculated as in Section 6.5.5, it is expected for the powers of the signals to be decreasing while the signals travel through the medium. The amount of loss in the signals’ powers depends on the amount of the noise power and the attenuation present in the medium. Thus, the power of the received signal $P_{rx}$ can be calculated as

(18) $$P_{rx}\left(f,d\right)=\frac{S_{p.s.d}\left(f\right)}{A_{\mathrm{Total}}\left(f,d\right)\times P_{N}\left(f,d\right)}$$

where *f* the utilized frequency, *d* is the distance between the communicating NSs, $S_{p.s.d}$ is the power signal density, $A_{\mathrm{Total}}$ is the attenuation of the Terahertz signal, and $P_{N}$ is the power of the noise.

**Figure 11:**
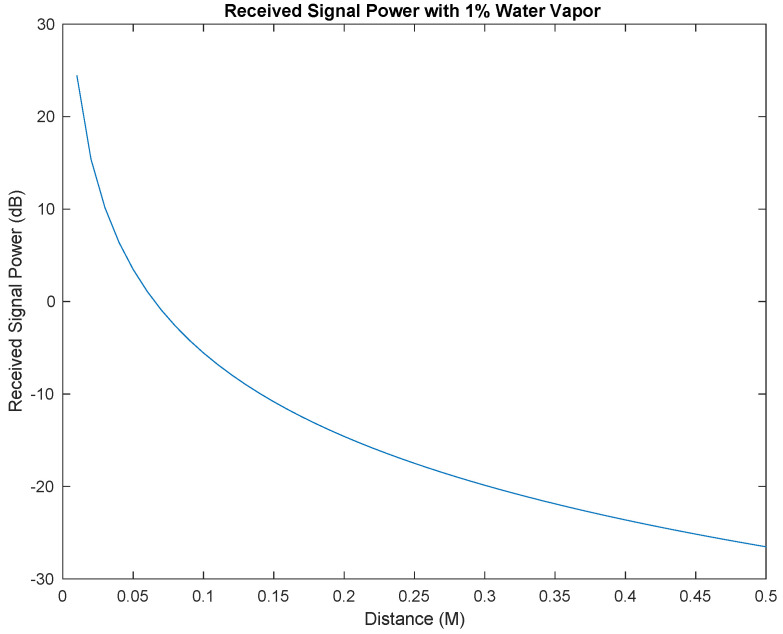
Received signal power.

Figure 11 shows the values of the received signal power for distances between 0.01 m and 1 m in a medium with 1% water vapor. As expected, the power of the received signal decreases significantly with the increasing in the distance between the communicating NSs.

In summary, the results presented in Figure 8, Figure 9, Figure 10 and Figure 11 highlight the fundamental physical constraints that govern the feasibility of deployment strategies in electromagnetic WNSNs. Figure 8, Figure 9, Figure 10 and Figure 11 demonstrate the severe propagation limitations and constraints of the THz band through attenuation, molecular absorption, and received power degradation. Although these behaviors follow established channel models, their visualization is crucial because they define the operational boundaries within which any deployment solution must function. By linking these physical effects to deployment decisions such as node placement, topology selection, and communication range planning, these figures collectively provide a clear rationale for why WNSNs require ultra-dense layouts, short-range links, and environment-aware design. Thus, this section synthesizes the key propagation characteristics that form the basis for any practical deployment strategies.

### 6.6. Comparative Analysis of Deployment Metrics

To provide a clearer synthesis of prior research, this subsection presents a comparative overview of deployment-related metrics from representative electromagnetic WNSN studies. Table 3 summarizes notable proposals according to their deployment types, evaluation metrics (energy efficiency, coverage, and connectivity), and main findings.

Overall, energy efficiency, coverage, and connectivity are the most commonly optimized parameters in a deployment solution. Centralized models [55] tend to achieve superior connectivity through coordinated control, whereas distributed approaches [30,33] demonstrate stronger adaptability in dynamic nanoscale environments. Hybrid and fuzzy-based schemes [50] offer balanced trade-offs between stability and energy efficiency, though they require greater computational overhead. Notably, 3D deployment models [47] improve volumetric coverage in biological contexts such as intrabody sensing, while dynamic infrastructure-based solutions [56] enhance routing reliability.

However, the lack of unified benchmarking metrics limits direct quantitative comparison among studies. Hence, this comparative analysis focuses on relative performance trends concluded from some of the cited research works.

This comparative analysis highlights the trade-offs between energy conservation, computational complexity, and coverage efficiency across various WNSN deployment paradigms. Centralized designs offer robust communication at the expense of flexibility, whereas distributed models demonstrate greater adaptability under constrained nanoscale conditions. Coverage, which is critical to achieving the intended objectives of WNSN applications, remains under-studied. Consequently, a standardized evaluation framework is essential to enable fair comparison of deployment strategies and to quantify performance in realistic nanoscale environments.

## 7. Conclusions

Finally, deploying WNSN is an essential topic in researching and studying nanotechnology. The deployment attributes are very much dependent on the requirements of the applications that the network is deployed for. There are several deployment decisions affected by the applications’ requirements such as the following:

- The extent of the WNSN coverage; the coverage of the area could either be full coverage for the entire deployment field or partial coverage, where only covering a part of the field can achieve the application’s mission.
- The type of coverage required for the target area, whether a 1-coverage is enough or k-coverage is required to ensure the fulfillment of the application’s mission.
- WNSN lifetime, whether the network is expected to have a perpetual lifetime which requires the utilization of the energy harvesting systems with a steady source of energy, or temporal lifetime where energy harvesting systems can depend on the existing energy sources in the medium.
- The placement of the NSs in the field, whether they start with fixed positions defined based on some attributes to guarantee the coverage and connectivity, or the deployment starts with a random positioning for NSs, in which case self-deployment for NSs is required.
- The mobility of the WNSNs, whether the NDs are required to move constantly in the environment, or once they reach their optimal positions they are required to maintain them until the application’s mission is completed.
- The dimensions in which the WNSN is deployed, whether it is a 2D environment where all NDs have the same height, or 3D environment where NDs are positioned with (x,y,z) coordinates.
- The topology of WNSN and the distances between NDs to guarantee the continuous connectivity between them.

Furthermore, WNSN deployment must consider the constraints of NSs, graphene antenna, Terahertz band peculiarities, limited energy and so on, to produce a stable network that is capable of achieving the application’s goals. A stable WNSN offers sufficient coverage of the targeted area and continuous connectivity between NSs.

### Future Work and Research Directions

While this review has focused on synthesizing theoretical and simulation-based studies related to WNSN deployment, significant opportunities remain for practical validation. Future research should emphasize the development of comprehensive simulation frameworks and nanoscale prototypes to verify the assumptions underlying current deployment and communication models. In particular, simulation-based validation of terahertz (THz) propagation and molecular absorption effects will be crucial to bridge the gap between theoretical modeling and real-world implementation. Experimental prototyping using emerging nanofabrication techniques could further substantiate the feasibility of the proposed architectures and enable the calibration of THz communication models. Such empirical studies would provide the foundation for transforming conceptual WNSN designs into deployable nanoscale systems.

In addition, future work should also explore emerging domains such as quantum-enhanced nanosensing and nanoscale security protocols, which are expected to play a significant role in reliable and secure WNSN deployment.

## Figures and Tables

**Table 1:** Comparison between sensors and nanosensors.

Item	Sensor	Nanosensor
Size	Vary from the size of a shoe box to the size of a grain of dust	10–100 μm${\,}^{2}$
Communication	Radio frequency (at 915 MHz and more recently 2.4 GHz)	Terahertz Band (0.1–10 THz)
Propagation Loss	Acceptable error rate	Very high propagation loss
Sensing Range	20 m to 30 m for indoor and 75 m to 100 m for outdoor	3 μm
Transmission Range	300 m for 900 MHz and 64 m for 2.4 GHz	Depends on the medium conditions, 0.03 m in medium with 1% water vapor using $10^{-18}$ J

**Table 2:** Classifying papers based on deployment’s attributes.

		Centralized Topology		Distributed Topology				
Paper	Key Concept	Cluster	Infrastructure	Mesh	Mobile NSs	Static NSs	2D Environment	3D Environment
[13]	Proposes EM-based WNSN architectures and discusses deployment considerations	x				x		
[30]	PSO-based mobility control to extend WNSN lifetime				x	x		x
[44]	Introduces scalable nanomachine architecture applicable to deployment planning			x				
[45]	Proposes SDM-based WNSN architecture influencing topology selection			x			x	
[46]	Biological communication model relevant for nanoscale deployment					x	x	
[47]	Evaluates THz propagation in plant monitoring deployments	x				x		x
[49]	Graphene-grid placement for energy-efficient WNSN			x	x		x	
[50]	Fuzzy-logic mobility management for WNSNs		x		x		x	
[51]	3D deployment model for in-body sensing		x		x			x
[52]	AI-Driven Nano-Router Localization in WNSNs	x			x	x	x	
[53]	PSO-driven self-deployment to improve coverage	x						
[54]	Hop-count localization applicable to distributed deployment			x		x	x	
[55]	Forwarding mechanisms for EM WNSNs	x				x	x	
[56]	Routing and coordinates system influencing topology	x		x		x	x	
[57]	Modulation strategy for better THz nanonetwork performance			x		x		x

**Table 3:** Comparative summary of deployment metrics in WNSN studies.

Reference	Deployment Type/Topology	Energy Efficiency	Coverage Ratio	Connectivity Performance	Key Observations
[30]	Distributed (PSO-based Mobility Control)	High—improved network lifetime	Improved-simulations	Stable under mobility	Effective for coverage and lifetime optimization, though computationally heavy.
[33]	Distributed Self-Deployment (PSO with Coverage Criterion)	Moderate—energy not prioritized	Full area coverage (k-covered)	Stable, adaptive	Strong self-organization; energy ignored.
[47]	Static Clustered 3D Deployment	Moderate—dependent on THz loss modeling	Not specified	Reliable	Suitable for plant and environmental monitoring.
[55]	Centralized Channel-Aware Forwarding	High—optimized transmission control	Not specified	Excellent; deterministic	Reliable but requires location awareness.
[56]	Distributed Dynamic Infrastructure (CORONA)	High—reduced retransmissions	Not specified	High; topology-aware	Improves routing reliability and link persistence.
[50]	Hybrid (Fuzzy Logic-based Mobility)	Moderate—depends on NC selection	Not specified	Stable; adaptive	Balanced load and link stability with fuzzy logic control.
[52]	AI-Driven/Mesh	Improved by shorter communication paths	98.03%	Enhanced connectivity by ensuring nodes are within range of a nano-router	Highlights the potential of AI/ML strategies to address deployment challenges in dense nanoscale networks.

## Data Availability

Data sharing is not applicable.
